# Metachronous isolated breast metastasis from pulmonary adenocarcinoma with micropapillary component causing diagnostic challenges

**DOI:** 10.1186/1471-2407-14-736

**Published:** 2014-10-01

**Authors:** Young Ju Jeong, Jin Gu Bong, Hoon Kyu Oh, Sung Hwan Park, Sung Min Kang, Sung Hwa Bae

**Affiliations:** Department of Surgery, Catholic University of Daegu School of Medicine, 33, Duryugongwon-ro 17-gil, Nam-gu, Daegu, Korea; Department of Pathology, Catholic University of Daegu School of Medicine, 33, Duryugongwon-ro 17-gil, Nam-gu, Daegu, Korea; Department of Nuclear medicine, Catholic University of Daegu School of Medicine, 33, Duryugongwon-ro 17-gil, Nam-gu, Daegu, Korea; Department of Internal Medicine, Catholic University of Daegu School of Medicine, (705-718) 33, Duryugongwon-ro 17-gil, Nam-gu, Daegu, Korea

**Keywords:** Breast metastasis, Pulmonary adenocarcinoma, Micropapillary component, Lung cancer

## Abstract

**Background:**

Breast metastasis from extramammary malignancy is uncommon and often presents diagnostic challenges. Herein, we report a case of a patient with metachronous isolated breast metastasis from pulmonary adenocarcinoma with micropapillary component.

**Case presentation:**

A 47-year-old woman presented with left breast nodule detected on a screening breast ultrasonography. She had surgery for pulmonary adenocarcinoma 3 years ago, and was disease-free state in the follow up studies. The patient was diagnosed with invasive micropapillary carcinoma of the breast by core needle biopsy. She underwent left breast lumpectomy and sentinel lymph node biopsy, and the histologic findings revealed micropapillary carcinoma. Based on the immunohistochemical study, the final diagnosis was solitary breast metastasis from pulmonary adenocarcinoma with micropapillary component.

**Conclusions:**

The diagnosis of metastasis to the breast from extramammary malignancies is difficult but important for proper management and prediction of prognosis. A careful clinical history with a thorough clinical examination is needed to make the correct diagnosis.

## Background

Metastases to the breast from extramammary malignancy are relatively rare though breast cancer is the most common malignancy in women [1]. Most malignancies seen in the breast are primary carcinomas [2] and the incidence of metastatic disease to the breast is 0.2-6.6% of all malignant breast tumors [3–8]. The primary malignancies most commonly metastasizing to the breast are leukemia, lymphoma, malignant melanoma and carcinomas from the lung, genitourinary or gastrointestinal tract [9–11]. These metastatic tumors can morphologically simulate breast cancer and lead to misclassification [11], which causes clinical problems because the treatment and prognosis of each tumor differs significantly.

Although lung cancer is one of most common cancer worldwide, there have been only a few published cases of pulmonary carcinoma metastasizing to the breast, particularly with micropapillary component [12–14]. Invasive micropapillary carcinomas have been described in several organs including urinary bladder, lung, major salivary glands, ovary and breast [15]. Adenocarcinoma with micropapillary component is a morphologic variant of carcinoma and usually recognized as a poor prognostic predictors [15]. We report a case of metachronous isolated breast metastasis from pulmonary adenocarcinoma with micropapillary component which was initially favored to be primary breast cancer. The institutional review board at Daegu Catholic University Hospital granted an exemption from requiring ethics approval for this study.

## Case presentation

A 47-year-old Korean woman presented to the Daegu Catholic University Hospital in Korea with a nodule revealed by screening breast ultrasonography in her left breast. On the physical examination, there was no palpable mass or nipple discharge in both breasts. There was no clinical evidence of regional lymphadenopathy. She had no family history of breast or ovarian cancers. She was a non-smoker but had left upper lobectomy of lung because of a pulmonary adenocarcinoma 3 years ago. The pathologic diagnosis was 3.5 cm-sized adenocarcinoma with micropapillary component, and the pathologic stage of lung cancer was T2aN0M0, stage IB.

**Figure 1:**
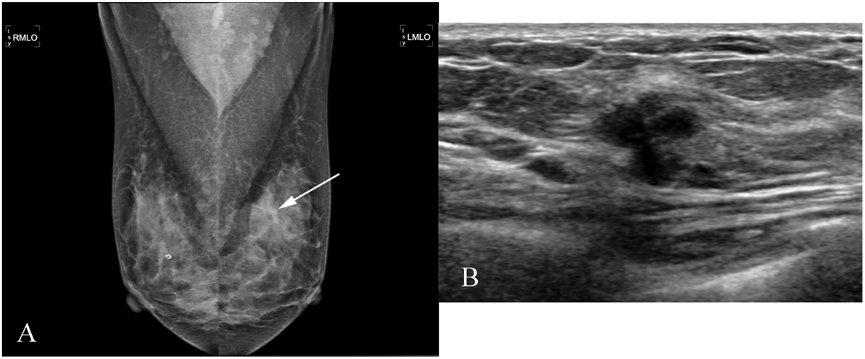
**Radiologic findings of the left breast. (A)** Mammography showing a focal asymmetry in left upper breast and benign calcifications in both breasts **(B)** Ultrasonography showing irregular shaped and microlobulated hypoechoic small mass in left upper breast.

**Figure 2:**
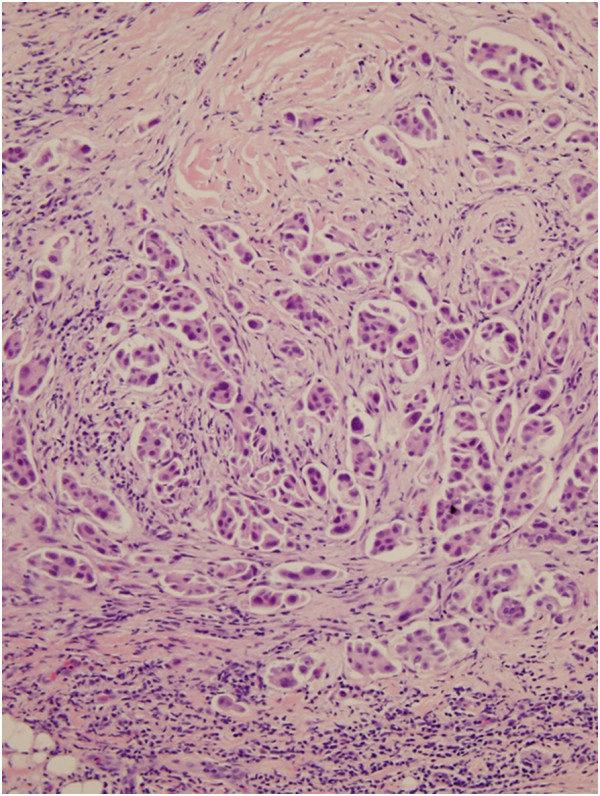
**Core needle biopsy of left breast tumor.** Microscopic findings of the specimen showed proliferation of micropapillae of anaplastic cells in the clear spaces which were consistent with invasive micropapillary carcinoma of the breast (H&E stain, ×400).

Mammography revealed only a focal asymmetry in left upper breast and benign calcifications in both breasts (Figure 1A). Ultrasonography revealed two irregular shaped and microlobulated hypoechoic small masses in left upper breast, which was categorized according to Breast Imaging Report and Data System (BI-RADS) as BI-RADS 4C (Figure 1B). The patient underwent ultrasound-guided core needle biopsy. Initial histologic findings revealed proliferation of micropapillae of anaplastic cells in the clear spaces which were consistent with invasive micropapillary carcinoma of the breast (Figure 2). Magnetic resonance imaging of the breast showed no significantly enhancing lesion in both breast. Chest computed tomography (CT) revealed probable benign sub-pleural nodule in right lower lobe but no tumor recurrence or nodal metastasis of lung cancer. On a position emission tomography-CT image there was no evidence of tumor recurrence of lung cancer or distant metastasis.

**Figure 3:**
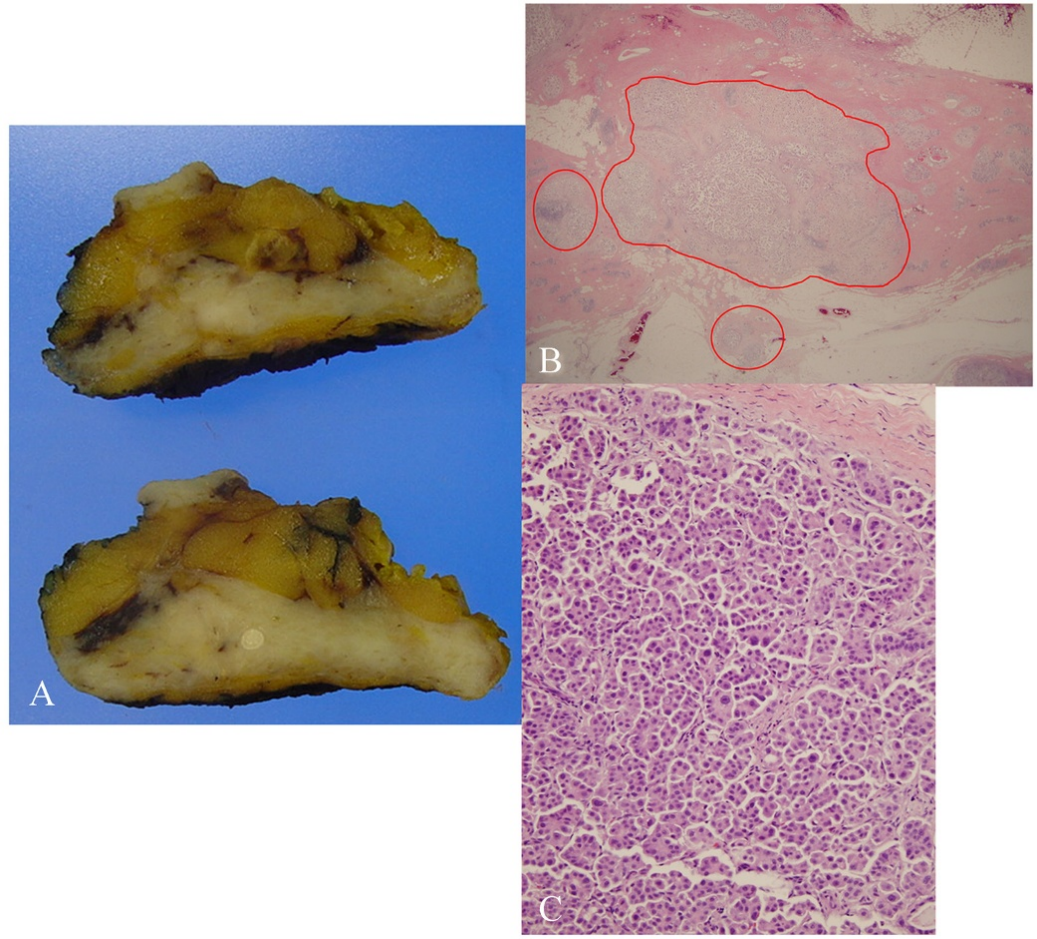
**Histologic findings of the left breast tumor after lumpectomy. (A)** Gross findings of the specimen showed an irregular shaped whitish fibronodular lesion. **(B)** Hematoxylin and eosin (H&E)-stained paraffin sections of the lumpectomy specimen revealed extensive micropapillary components (H&E stain, ×40). **(C)** Microscopic findings of the specimen showed small clusters of cells within clear stromal spaces resembling dilated vascular channels with a desmoplastic reaction (H&E stain, ×400).

**Figure 4:**
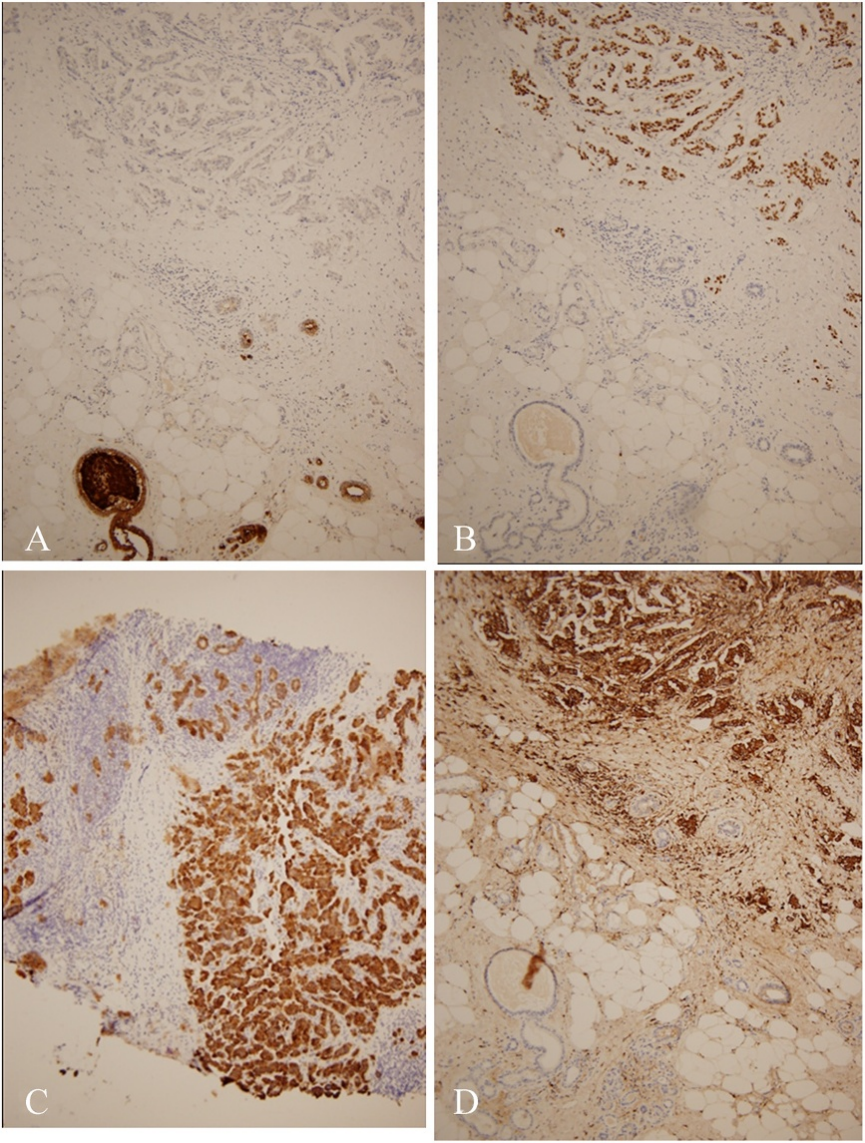
**Immunohistochemical staining of the left breast tumor after lumpectomy. (A)** GCDFP-15 stain was negative in malignant cells (×400). **(B)** TTF-1 stain reveals nuclear positivity (×400). **(C)** CK-7 stain was positive in malignant cells (×400). **(D)** Napsin A stain was positive in malignant cells (×400).

Clinically, primary breast cancer was suspected, and the patient underwent lumpectomy and sentinel lymph node biopsy using radio-isotope and indigocarmine dye. Gross examination of the specimen revealed an irregular shaped whitish fibronodular lesion, measuring 1.3 × 1 cm in size (Figure 3A). The specimen was fixed in 10% formalin, and paraffin sections were prepared. Hematoxylin and eosin (H&E)-stained paraffin sections of the lumpectomy specimen revealed extensive micropapillary components (Figure 3B). Microscopic examination of the sections from the specimen showed small clusters of cells within clear stromal spaces resembling dilated vascular channels (Figure 3C) with a desmoplastic reaction. There were no ductal components and carcinoma in situ lesions. There were lymphovascular invasion and some microscopic multiple tumor foci, but resection margins were free from tumor. The tumor cells showed triple negative immunoreactivity for estrogen receptor (ER), progesterone receptor (PR) and HER2/neu. The immunohistochemical staining for gross cystic disease fluid protein-15 (GCDFP-15) (Figure 4A) and anaplastic lymphoma kinase (ALK) was negative, but, that for thyroid transcription factor-1 (TTF-1), cytokeratin-7 (CK-7) and Napsin A was positive (Figure 4B-4D). There was no axillary lymph node metastasis. Based on the histology and the immunohistochemical staining patterns, this breast tumor was supposed to be a metastatic adenocarcinoma from pulmonary malignancy.

We reviewed the histopathologic findings of the lung cancer removed 3 years ago and compared them with the findings of the breast tumor. H&E-stained paraffin sections of the lung cancer revealed diffuse infiltration of malignant epithelioid cells showing solid and micropapillary patterns, which resemble the findings of the breast tumor. Also, the lung cancer had the same immunoprofiles as the breast tumor.

**Figure 5:**
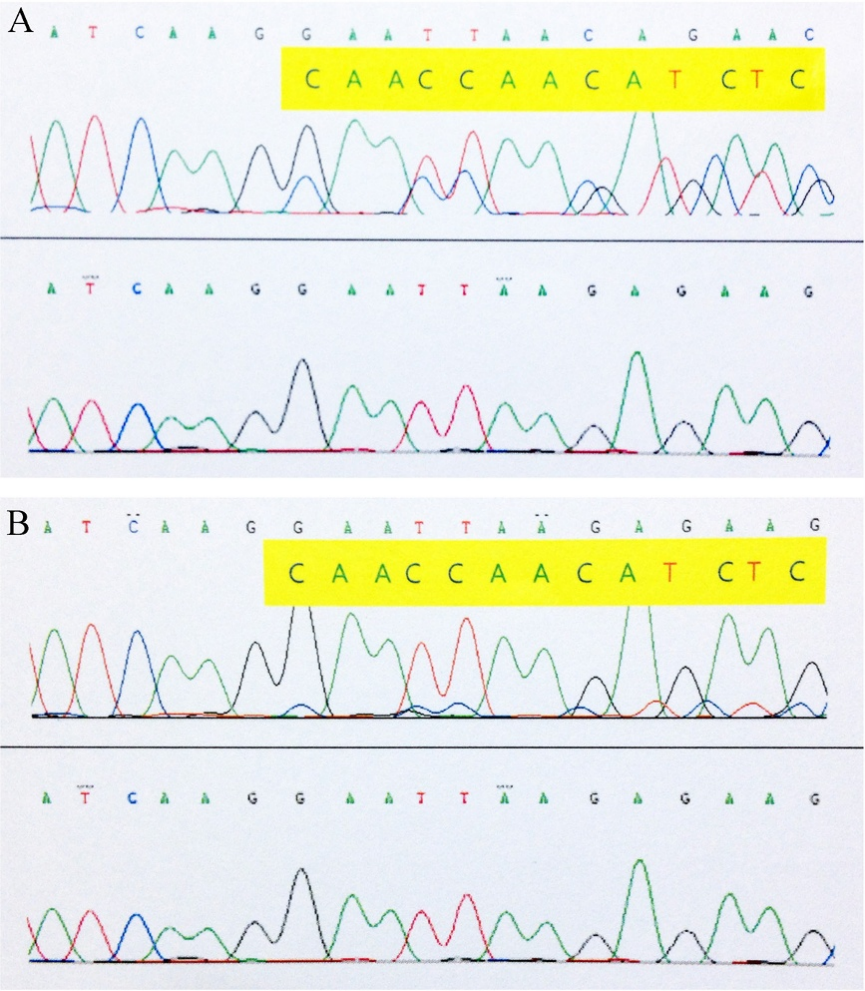
**Sequencing results of the heterozygote mutations c.2239_2247del9 (p.L747_E749del) of** ***EGFR*** **gene in breast tumor specimen (A) and lung adencarcinoma specimen (B).**

We analyzed the mutation of *EGFR* gene in the breast tumor and the original pulmonary adenocarcinoma specimen. Once informed consent had been obtained, genomic DNA was extracted from paraffin-embedded tumor specimens using QIAamp DNA FFPE Tissue kit (Qiagen, Hilden, Germany) following the manufacturer’s instructions. Polymerase chain reaction and mutational analyses of the genes were performed. Exon 18, 19, 20 and 21 of the *EGFR* gene were analyzed by direct sequencing. DNA sequencing was performed on the pretreated PCR product using an automated direct sequence analyzer (ABI PRISM 3100 Genetic Analyzer; Applied Biosystems, Foster City, CA, USA). The mutationl analysis revealed same heterozygote mutation in both of the specimens. There was a 9-bp deletion in exon 19, namely, c.2239_2247del9, which resulted in a deletion of three amino acids, namely p.L747_E749del (Figure 5).

From the above results, the patient was diagnosed solitary breast metastasis from pulmonary adenocarcinoma with micropapillary component. Since the mutation test for *EGFR* was positive, the patient has been taking Gefitinib, being disease-free at 23 month after the diagnosis of the breast metastasis.

## Conclusions

The breast is an unusual site for metastasis from extramammary tumors and metastatic cancer is an unexpected diagnosis in a woman presenting with a breast mass [16, 17]. The distinction between breast metastasis from extramammary malignancy and primary breast cancer is important for patient management [11]. Some authors have described clinical and histological characteristics of breast metastasis from extramammary malignancies [5, 8, 11, 18]. The most common symptoms are solitary discrete lesions in the breast [5, 11], while in one study, most patients presented with a history of loco-regional and wide spread metastases of extramammary neoplasms [18]. The most common mammographic appearance is of a round mass with well-defined or slightly irregular margins [11, 18]. In our patient, mammography revealed only a focal asymmetry in left upper and ultrasonography revealed two irregular shaped and microlobulated hypoechoic small masses in left upper breast categorized according to BI-RADS 4C. Histological features of metastases to the breast include atypical histologic features for a primary breast carcinoma, a well-circumscribed tumor with multiple satellite foci, the absence of an intraductal component, and the presence of many lymphatic emboli [4]. However, pathologic diagnosis of breast metastases is difficult, because many extramammary malignancies lack specific histological features and sometimes the features are similar to those of primary breast cancer, particularly with extensive micropapillary patterns [12, 13].

Clinical history, radiologic findings and histologic features are helpful in the evaluation of metastatic lesions in the breast. In a review of the ultrasonographic appearances of breast metastases from extramammary malignancies, there are several typical features of breast metastases [19]. Typical ultrasound features of hematogenous metastases include single or multiple, round to oval shaped, well-circumscribed hypoechoic masses without spiculations, calcifications, or architectural distortion [19]. However, lesions show variable radiologic features in some cases and a possibility of a metastasis should be suspected for a breast tumor in a patient with a history of cancer, even if clinically or radiologically benign [20]. Most of primary breast carcinomas originate in the ducts or lobules of the breast and the presence of an in situ (intraductal) component is the only absolute proof of the primary breast carcinoma [21]. After all, whenever a well-circumscribed tumor is identified in the breast showing lack of in situ components, the possibility of metastatic cancer should be considered and excluded [21], especially in high grade tumors without an in situ component. Also, ER and PR are highly specific markers for breast cancer [11] and ER/PR negative breast tumor without an in situ component is the most common clue for suspicion of metastatic tumor in the breast.

Immunohistochemical studies are necessary for pathologic diagnosis if no primary tumor was known and the clues are subtle to show specific histological features [4, 11]. An immunohistochemical analysis using a panel of antibodies may be useful to discriminate a primary mammary tumor from an extramammary malignancy because specificity or sensitivity of specific markers is not always 100% [11]. The combination CK7 and CK20 is useful in categorizing carcinomas [11, 22]. The most of breast carcinomas are CK7+ and CK20-, and a CK20+ or CK7- pattern would make breast origin less likely [22]. GCDFP-15 is also highly specific marker for breast cancer [11]. TTF-1 is a very useful marker in distinguishing pulmonary adenocarcinomas from other primary carcinomas. TTF-1 is expressed in about 75% of pulmonary adenocarcinomas [11], and no breast carcinomas have been reported to be positive for TTF-1 except rare small cell carcinomas of the breast [23, 24]. Napsin A is a new marker for pulmonary adenocarcinoma and is known to be more sensitive and specific than TTF-1 in the differential diagnosis of primary pulmonary carcinoma [25]. In our case, the tumor cells showed negative immunoreactivity for ER, PR, HER2/neu and GCDFP-15, and positive for TTF-1, CK-7 and Napsin A.

In a systematic review of the literature, 43 independent case reports were identified for primary lung cancer metastasis to the breast [26]. Of these 43 case reports, only 3 case reports revealed metastasis to the breast from pulmonary adenocarcinoma with a micropapillary component (Table 1). These 3 previous reports [12–14] have described synchronous lung cancer with breast metastasis and demonstrated similar histologic features of breast biopsy including adenocarcinoma with micropapillary component, lymphovascular invasion (lymphatic tumor emboli) and desmoplastic reaction such as dense fibrohyalinized stroma. Maounis et al. [13] and Sanguinetti et al. [14] and also described multiple psammoma bodies in tumor. In our case, adenocarcinoma with micropapillary component, desmoplastic reaction and lymphovascular invasion were identified, but psammoma body was not observed. To the best of our knowledge this is the first report of metachronous breast metastasis from pulmonary adenocarcinoma with micropapillary component.

**Table 1:** **Clinical features of case reports of breast metastasis from pulmonary adenocarcinoma with micropapillary components**

Authors, year	Age/Sex	Chief complaint	Method of detection for breast tumor	Breast tumor size	Metachronous	Initial stage of lung cancer	Management	Chemotherapy regimen	Survival	Follow-up
Ko K, et al., 2012 [12]	47/F	Chest pain with dyspnea	Palpable mass on P/Ex.	1 cm in diameter	No	IV	Chemotherapy	Cisplatin/Irinotecan followed by erotinib	Alive	8 mo
Maounis N, et al., 2010 [13]	73/F	Dyspnea with dry cough	Palpable mass on P/Ex	Not available	No	IV	Chemotherapy	Cisplatin/docetaxel/bevacizumab	Dead	6 mo
Sanguinetti A, et al., 2013 [14]	43/F	Dyspnea with dry cough	Palpable mass on P/Ex	Not available	No	IV	Simple mastectomy + Chemotherapy	Cisplatin/docetaxel/bevacizumab	Dead	8 mo
This report	47/F	Breast nodule on screening exam	Ultrasonography	1.3 cm ×1 cm	Yes	IB	Lumpectomy + Chemotherapy	Gefitinib	Alive	23 mo

F, female; P/Ex, physical examination.

It has been documented that breast metastasis from extramammary malignancy has a poor prognosis because most patients have been reported as widely disseminated disease and die within a year of diagnosis [10, 11]. Specifically, synchronously-presenting lung cancer metastasizing to the breast, namely stage IV lung cancer has carried a very poor prognosis [26]. For patients presenting synchronous lung cancer with breast metastasis, removal of the breast lesion offers no patient benefit [26]. However, removal of the breast lesion could be a useful treatment option for patients with metachronously-presenting lung cancer metastasizing to the breast only, although it has not been established yet whether surgical treatment will affect the prognosis or not. In our case, the patient had only metastasis to the breast and was treated with surgery and anti-EGFR (Gefitinib) treatment. She has been survived with disease free for 23 months following the diagnosis of the breast metastasis.

Micropapillary component is generally thought to have prognostic significance and is associated with a manifestation of aggressive behavior such as lymph node metastases and distant metastases [27, 28]. In 3 previous reports [12–14] of synchronously-presenting breast metastasis from pulmonary adenocarcinoma with a micropapillary component, 2 patients died 6 months and 8 months following diagnosis, respectively [13, 14], and a patient were alive 8 months after the initial diagnosis of lung cancer [12], although all patients have received systemic chemotherapy. Our patient is currently alive without additional metastasis 23 months after the diagnosis of the breast metastasis, but long-term follow-up is needed.

Here, we report a rare case of metachronous isolated metastasis to the breast from a pulmonary adenocarcinoma with micropapillary component. The distinction between breast metastasis from pulnomary adenocarcinoma with micropapillary component and primary breast micropapillary carcinoma may cause diagnostic challenges. An immunohistochemical analysis is useful for accurate diagnosis. Furthermore, although it is rare, the possibility of metastatic disease to the breast should be considered before making the diagnosis of primary breast cancer, particularly with micropapillary component.

## Consent

Written informed consent was obtained from the patient for publication of this case report and accompanying images. A copy of the written consent is available for review by the Editor of this journal.
